# Aloe-emodin inhibits HER-2 expression through the downregulation of Y-box binding protein-1 in HER-2-overexpressing human breast cancer cells

**DOI:** 10.18632/oncotarget.10410

**Published:** 2016-07-06

**Authors:** Jui-Wen Ma, Chao-Ming Hung, Ying-Chao Lin, Chi-Tang Ho, Jung-Yie Kao, Tzong-Der Way

**Affiliations:** ^1^ Institute of Biochemistry, College of Life Science, National Chung Hsing University, Taichung, Taiwan; ^2^ Department of General Surgery, E-Da Hospital, I-Shou University, Kaohsiung, Taiwan; ^3^ School of Medicine, I-Shou University, Kaohsiung, Taiwan; ^4^ Division of Neurosurgery, Buddhist Tzu Chi General Hospital, Taichung Branch, Taiwan; ^5^ School of Medicine, Tzu Chi University, Hualien, Taiwan; ^6^ Department of Medical Imaging and Radiological Science, Central Taiwan University of Science and Technology, Taichung, Taiwan; ^7^ Department of Food Science, Rutgers University, New Brunswick, New Jersey, USA; ^8^ Department of Biological Science and Technology, College of Biopharmaceutical and Food Sciences, China Medical University, Taichung, Taiwan; ^9^ Department of Health and Nutrition Biotechnology, College of Health Science, Asia University, Taichung, Taiwan

**Keywords:** HER-2-overexpressing breast cancer cells, aloe-emodin, Y-box binding protein-1, ILK/Akt/mTOR signaling pathway, epithelial-mesenchymal transition

## Abstract

Human epidermal growth factor receptor-2 (HER-2)-positive breast cancer tends to be aggressive, highly metastatic, and drug resistant and spreads rapidly. Studies have indicated that emodin inhibits HER-2 expression. This study compared the HER-2-inhibitory effects of two compounds extracted from rhubarb roots: aloe-emodin (AE) and rhein. Our results indicated that AE exerted the most potent inhibitory effect on HER-2 expression. Treatment of HER-2-overexpressing breast cancer cells with AE reduced tumor initiation, cell migration, and cell invasion. AE was able to suppress YB-1 expression, further suppressing downstream HER-2 expression. AE suppressed YB-1 expression through the inhibition of Twist in HER-2-overexpressing breast cancer cells. Our data also found that AE inhibited cancer metastasis and cancer stem cells through the inhibition of EMT. Interestingly, AE suppressed YB-1 expression through the downregulation of the intracellular integrin-linked kinase (ILK)/protein kinase B (Akt)/mTOR signaling pathway in HER-2-overexpressing breast cancer cells. *In vivo* study showed the positive result of antitumor activity of AE in nude mice injected with human HER-2-overexpressing breast cancer cells. These findings suggest the possible application of AE in the treatment of HER-2-positive breast cancer.

## INTRODUCTION

Breast cancer is the most common cancer among women worldwide [1]. Among the clinical classifications of breast cancer [2], human epidermal growth factor receptor-2 (HER-2)-overexpressing breast cancer has a high risk of treatment failure because it spreads rapidly and is highly metastatic as well as drug resistant [3]. Clinically, Herceptin is the most common treatment [4]; however, some patients exhibit drug resistance within 1 year of treatment [5]. Therefore, overcoming drug resistance and establishing more effective treatment options for patients with HER-2 overexpression is crucial [6].

Epithelial–mesenchymal transition (EMT) is a key event in cancer metastasis [7]. Tumor hypoxia results in the high expression of hypoxia-inducible factors (HIFs), thereby inducing EMT [8]. After EMT induction, the transcription factors Snail or Twist suppresses the intercellular binding protein E-cadherin, reducing binding between cancer cells and generating highly aggressive and mobile cancer cells; this promotes the departure of cancer cells from the primary tumor, leading to tumor metastasis [9]. EMT can induce the generation of cancer cells with stem cell characteristics [10], and cancer stem cells are one of the most crucial factors in cancer recurrence, metastasis, and drug resistance [11].

Y-box binding protein 1 (YB-1) is a multifunctional protein involved in transcription and translation; high YB-1 expression promotes cell proliferation and inhibits apoptosis, tumor invasion and metastasis, and angiogenesis [12]. Studies have found that YB-1 can promote breast cancer, bladder cancer, liver cancer, and progression and metastasis of other cancers [13] through EMT regulation. YB-1 expression, which has been detected in many cancers, promotes stem cell expression, improves mobility of cancer cells, and enhances expression of genes associated with drug resistance and other common characteristics of cancer stem cells [14–16].

Anthraquinones are a large group of natural aromatic compounds [17]. Such compounds are historically used as natural dyes, but recent studies demonstrated their medicinal values such as antibacterial, anti-inflammatory, antiviral, anticancer, and antiaging properties [18]. The anthraquinone emodin inhibits the proliferation of HER-2-overexpressing breast cancer cells [19] and induces apoptosis of cancer cells [20]. However, it is not known whether other anthraquinones, such as aloe-emodin (AE) and rhein, would suppress the proliferation of HER-2-overexpressing breast cancer cells. This study revealed that anthraquinones suppressed HER-2-overexpressing breast cancer cell proliferation; furthermore, the molecular changes during cell program death were explored.

## RESULTS

### Anthraquinone derivatives suppressed HER-2 expression and cell proliferation in HER-2-overexpressing breast cancer cells

**Figure 1:**
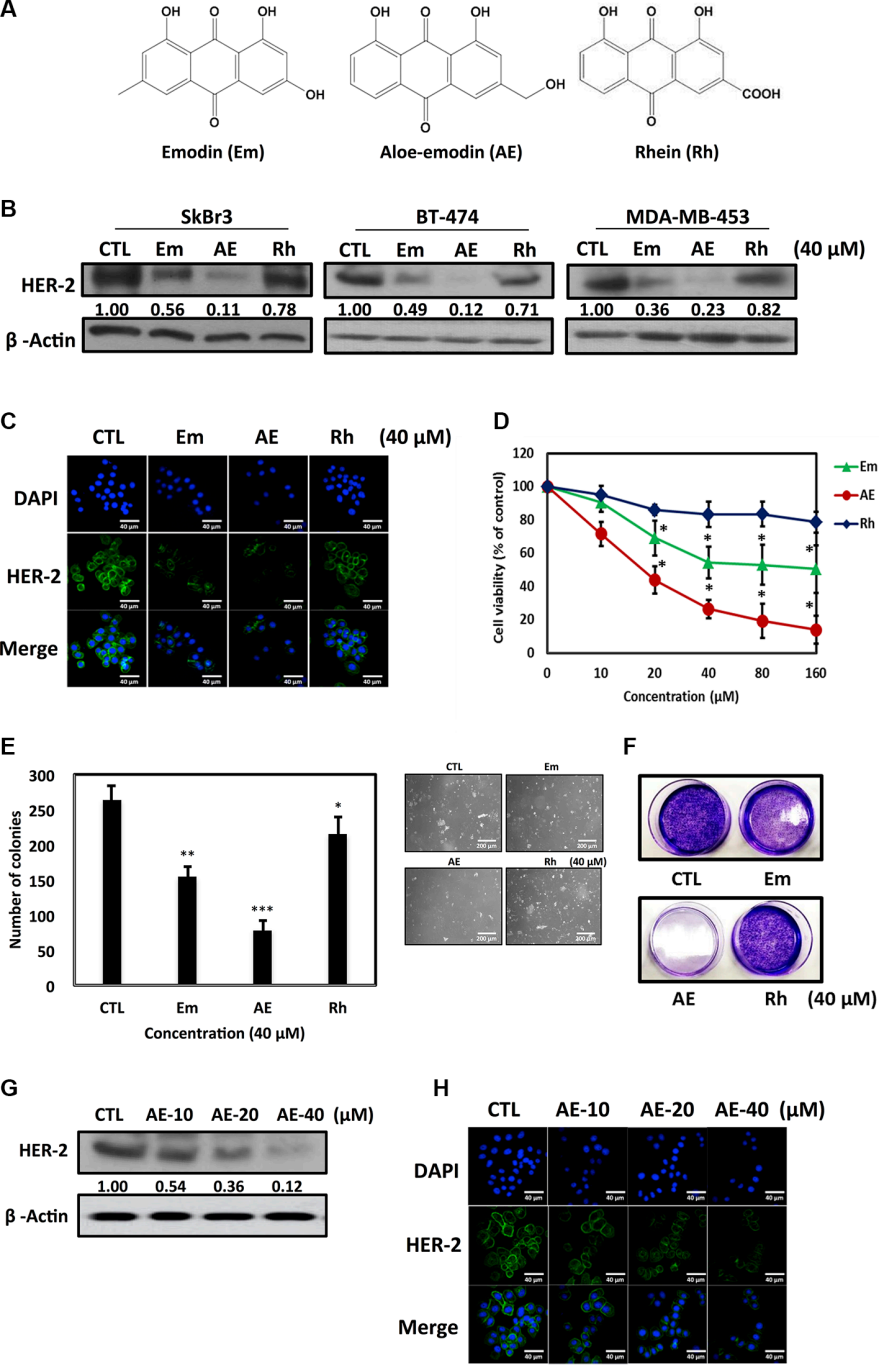
Effect of emodin, aloe-emodin and rhein on HER-2 expression in HER-2-overexpressing breast cancer cells (**A**) Structures of emodin (Em), aloe-emodin (AE) and rhein (Rh). (**B**) Cells were treated with 40 μM Em, AE and Rh for 48 h. Cell lysates were immunoblotted with anti-HER-2 antibody. β-Actin was used as the loading control. (**C**) Cells were treated with 40 μM Em, AE, and Rh for 48 h. HER-2 was visualized through immunofluorescence staining using anti-HER-2 antibodies and Alexa Fluor^®^-conjugated secondary antibodies (green). Labeling nuclear DNA using DAPI (blue). Scale bar, 40 μm. (**D**) Effect of Em, AE, and Rh on viability of HER-2 overexpressing breast cancer cells. The SkBr3 cell line was treated with various concentrations of Em, AE, and Rh at 37°C for 48 h. The effect on cell growth was examined using the MTT assay, and the percentage of cell viability was calculated by defining the absorption of cells without Em, AE, or Rh as 100%. (**E**) Anchorage-independent cell growth on soft agar. SkBr3 cells were grown on soft agar for 21 days in the presence of the 40 μM Em, AE, and Rh (20×). Statistical analysis of the experiment. Columns, mean (*n* = 5); bars, SD. (**F**) In the colony formation assay, SkBr3 cells were treated with 40 μM Em, AE and Rh. (**G**) Cells were treated with various concentrations of AE for 48 h. Cell lysates were immunoblotted with anti-HER-2 antibody. β-Actin was used as the loading control. (**H**) Immunofluorescence staining of HER-2 treated with various concentrations of AE. Each experiment was independently repeated three times (*n* = 3). The results are expressed as mean ± SD. *P < 0.05.

Studies have demonstrated that emodin, an anthraquinone compound, has antitumor effects in breast cancer [21]. In this study, we compared the inhibitory effects of three anthraquinones, emodin, AE, and rhein, (Figure 1A) on HER-2 expression in HER-2-overexpressing breast cancer cells. Among these three anthraquinones, AE had the highest inhibitory effect on HER-2 expression in SkBr3, BT-474, and MDA-MB-453 cell lines when they were treated with these compounds at a concentration of 40 μM for 48 h (Figure 1B). Immunofluorescence staining yielded similar results (Figure 1C). In a cell viability test, AE suppressed HER-2 expression in HER-2-overexpressing SkBr3 cell line (Figure 1D). In the soft agar assay, AE reduced the number of cells (Figure 1E). In the colony formation assay, AE had the highest inhibitory effect on the number of colonies, compared with emodin and rhein (Figure 1F). These findings showed that among anthraquinones, AE had the most significant suppressive effect on HER-2 expression and cell proliferation in HER-2-overexpressing breast cancer cells. Therefore, in subsequent experiments, we treated SkBr3 cells with different concentrations of AE (10, 20, and 40 μM) and observed the variation in the suppression of HER-2 expression. The Western blotting results showed that increasing AE therapeutic concentrations significantly and dose-dependently suppressed HER-2 expression (Figure 1G). Immunofluorescence staining also yielded similar results (Figure 1H). These results showed that compared with emodin and rhein, AE significantly suppressed HER-2 expression in HER-2-overexpressing breast cancer cells.

### AE specifically suppressed cell proliferation and induced apoptosis in HER-2-overexpressing breast cancer cells

**Figure 2:**
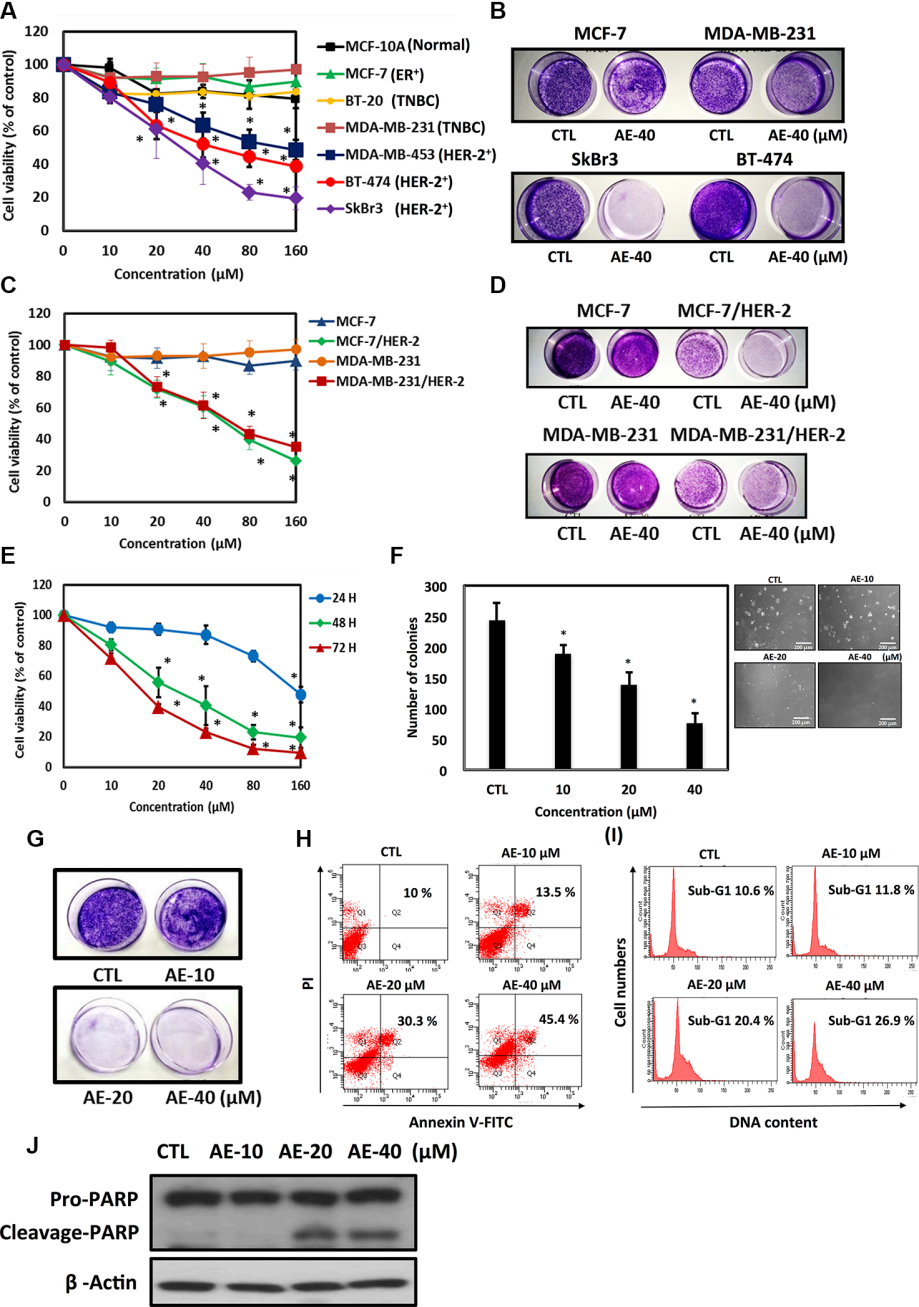
Aloe-emodin specifically inhibited cell proliferation and induced apoptosis in HER-2-overexpressing breast cancer cells (**A**) Effect of AE on the cells viability of different breast cancer cell lines. Different cell lines were treated with various concentrations of AE at 37°C for 48 h. The effect on cell growth was examined using the MTT assay. (**B**) In the colony formation assay, different breast cancer cell lines were treated with 40 μM AE. (**C**) In comparison with MCF-7, MDA-MB-231 and HER-2-transfected cells (MCF-7/HER-2 and MDA-MB-231/HER-2). Cell viability was determined using the MTT assay. (**D**) These cell lines were treated with the same experimental conditions for investigating colony formation in the colony formation assay. (**E**) Effect of AE on the viability of HER-2-overexpressing breast cancer cells. The SkBr3 cell line was treated with various concentrations of AE at 37°C for 24, 48, and 72 h. The effect on cell growth was examined using the MTT assay. (**F**) SkBr3 cells were treated with the same experimental conditions for anchorage-independent cell growth in soft agar. (**G**) In the colony formation assay, SkBr3 cells were treated with various concentrations of AE at 37°C for 48 h. (**H**) SkBr3 cells were treated with various concentrations of AE for 48 h. Cells were stained with PI and FITC-conjugated annexin V through flow cytometry. (**I**) Treatment of SkBr3 cells with various concentrations of AE for 48 h, and PI-stained DNA content was analyzed through flow cytometry. (**J**) SkBr3 cells were treated with various concentrations of AE for 48 h. Cells were then harvested and lysed for the detection of pro-PARP, cleaved PARP, and β-actin expression. Western blot data are representative of those obtained in at least three separate experiments. The results represent three repeated experiments and are expressed in mean ± SD. Each experiment was independent repeated three times (*n* = 3). *P < 0.05.

Among members of the epidermal growth factor receptor (HER, ErbB) family, HER-2 is the most potent oncogenic protein and positively correlates with the metastasis of cancer cells [22]. We next investigated whether AE specifically suppressed the proliferation of HER-2-overexpressing breast cancer cells. We used the MTT assay to examine the cell viability of different cell lines, including the estrogen receptor (ER)-positive, triple-negative breast cancer (TNBC), HER-2-overexpressing, and normal breast cell line, MCF-10A. After treatment with different concentrations of AE for 48 h, the results indicated that AE specifically suppressed the proliferation of HER-2-overexpressing cells (Figure 2A). The colony formation test also revealed the same result (Figure 2B). Furthermore, ER-positive and triple-negative breast cancer cells were transfected with HER2 to determine whether AE specifically suppresses the proliferation of HER-2-overexpressing cells. We used Western blotting to verify HER-2 overexpression in ER-overexpressing and triple-negative breast cancer cell lines (data not shown); moreover, we used the MTT test to compare cell transfection in HER-2-overexpressing and HER-2-non-overexpressing cell lines. We found that as the AE concentration increased, cell proliferation in the HER-2-overexpressing cell line decreased (Figure 2C). Moreover, the colony formation test yielded similar results (Figure 2D). The MTT assay revealed that AE treatment at different time points suppressed cell viability in SkBr3 cells (24 h, IC_50_ = 152.88 μM; 48 h, IC_50_ = 27.56 μM, 72 h, IC_50_ = 16.72 μM) (Figure 2E). The soft agar test showed that treatment with increasing concentrations of AE significantly reduced the number of colonies in SkBr3 cells (Figure 2F). In the colony formation assay, AE significantly reduced the number of colonies in SkBr3 cells (Figure 2G). Through Annexin V–PI double staining, we determined that AE induced apoptosis in SkBr3 cells (Figure 2H). In addition, we determined the effect of AE on cell cycle arrest in HER-2-overexpressing cells through flow cytometry. These results indicated that AE treatment for 48 h significantly induced sub-G1 cell cycle arrest in SkBr3 cells (Figure 2I). When cells undergo apoptosis, PARP in the nucleus is cleaved to form cleaved PARP. This study observed that treatment with increasing concentrations of AE significantly increased cleaved PARP (Figure 2J). It showed that AE treatment specifically suppressed proliferation of HER-2-overexpressing cells by inducing apoptosis.

### AE suppressed YB-1 expression in SkBr3 cells

**Figure 3:**
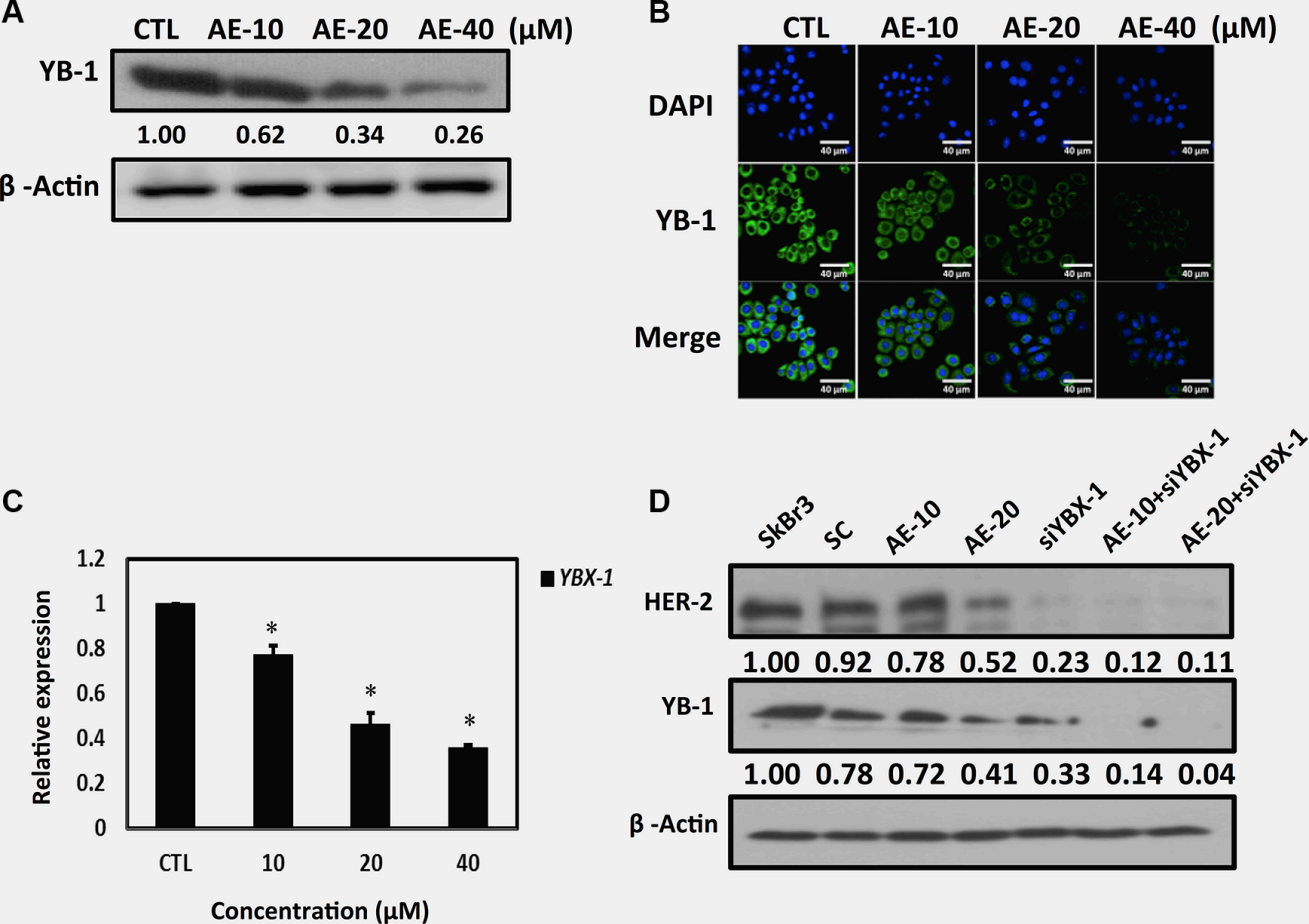
Aloe-emodin suppressed oncoprotein YB-1 expression in HER-2-overexpressing breast cancer cells (**A**) SkBr3 cells were treated with various concentrations of AE for 48 h. Cell lysates were immunoblotted with anti-YB-1 antibody. β-Actin was used as the loading control. (**B**) SkBr3 cells were treated with various concentrations of AE for 48 h. HER-2 was visualized through immunofluorescence staining using anti-HER-2 antibodies (green). Labeling nuclear DNA using DAPI (blue). Scale bar, 40 μm. (**C**) SkBr3 cells were treated with various concentrations of AE for 24 h. The YB-1 mRNA levels were examined using real-time qPCR. Data are expressed in mean ± SD. (**D**) SkBr3 cells were treated with various concentrations of AE for 48 h and transfected with YBX-1-siRNA in combination with treatment with various concentrations of AE. Cell lysates were immunoblotted with anti-HER-2 and anti-YB-1 antibodies. β-Actin was used as the loading control.

A clinical study showed that YB-1 overexpression was detected in patients with HER-2 overexpression [23], and it also promoted cell proliferation, tumor metastasis, invasion and angiogenesis [24]. In this study, we determined whether AE suppressed YB-1 expression in HER-2-overexpressing cell lines. The results showed that increasing concentrations of AE more strongly suppressed YB-1 expression (Figure 3A). Similar results were obtained through immunofluorescence staining (Figure 3B). Moreover, we determined whether AE reduced YB-1 expression through transcription. We used qRT-PCR to observe mRNA levels. Our results showed that increasing concentrations of AE significantly increased the suppression of YB-1 mRNA levels. The results confirmed that AE suppressed YB-1 during transcription, which resulted in lower protein expression (Figure 3C). We next used siRNA to examine whether silencing of YB-1 expression affected HER-2 expression. The results showed that silencing YB-1 expression reduced HER-2 expression. Interestingly, silencing YB-1 expression and treating with AE suppressed HER-2 expression (Figure 3D). These results showed that in HER-2-overexpressing cell lines, suppression of YB-1 expression by AE further suppressed downstream HER-2 expression.

### AE downregulated Twist expression in SkBr3 cells

**Figure 4:**
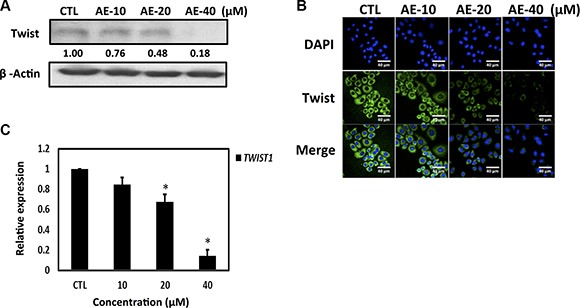
Aloe-emodin inhibited Twist expression in SkBr3 cells (**A**) SkBr3 cells were treated with various concentrations of AE for 48 h. Cell lysates were immunoblotted with anti-Twist antibody. (**B**) SkBr3 cells were treated with various concentrations of AE for 48 h. Twist was visualized by immunofluorescence staining using anti-Twist antibodies (green). (**C**) SkBr3 cells were treated with various concentrations of AE for 24 h. Twist mRNA levels were examined using real-time qPCR. Data are expressed the mean ± SD.

Studies have indicated that Twist regulates YB-1 expression and both Twist and YB-1 promote malignant potentials, including tumor growth, invasion and anti-cancer-drug resistance [25, 26]. We next determined whether AE suppressed YB-1 expression through the inhibition of Twist in HER-2-overexpressing breast cancer cells. Western blot results showed that increasing concentrations of AE significantly inhibited Twist expression (Figure 4A), and immunofluorescence staining yielded similar results (Figure 4B). We used qRT-PCR to investigate whether Twist mRNA levels changed after AE treatment. The results showed that increasing concentrations of AE significantly inhibited Twist mRNA levels (Figure 4C) and that AE treatment significantly inhibited the oncogenic transcription factor Twist through transcription. Our data indicated that treatment with AE suppressed YB-1 expression through the inhibition of Twist in HER-2-overexpressing breast cancer cells.

### AE inhibited epithelial-mesenchymal transition in SkBr3 cells

**Figure 5:**
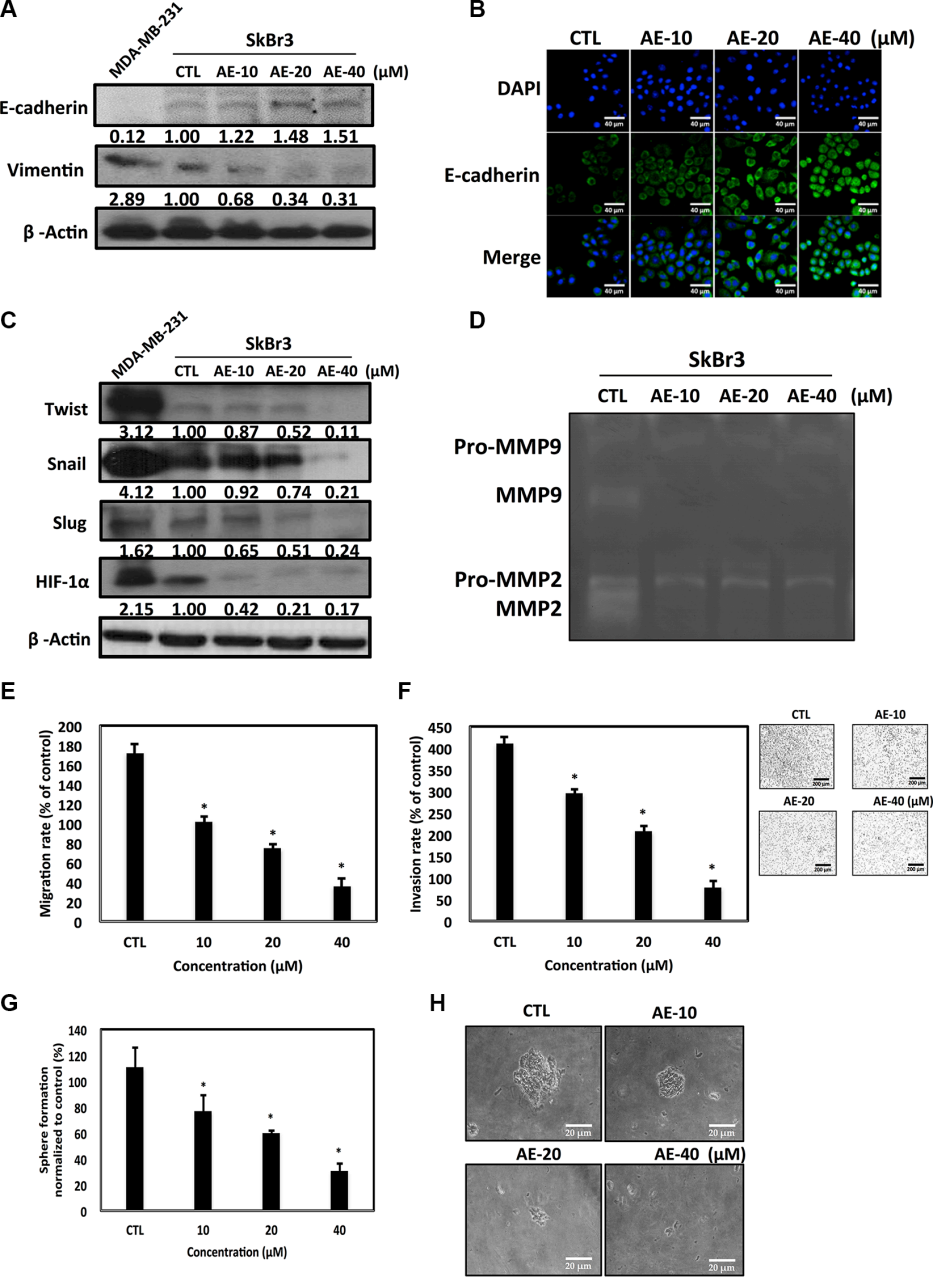
Aloe-emodin inhibited epithelial–mesenchymal transition in HER-2-overexpressing breast cancer cells (**A**) SkBr3 cells treated with various concentrations of AE for 48 h, the expression of epithelial cell marker E-cadherin and mesenchymal cell marker vimentin were assayed by Western blotting. (**B**) SkBr3 cells were treated with various concentrations of AE for 48 h. E-cadherin was visualized through immunofluorescence staining using anti-E-cadherin antibodies (green). (**C**) SkBr3 cells treated with various concentrations of AE for 48 h, the expression of transcription factor Twist, Snail, Slug, and HIF-1α were assayed by Western blotting. Values represent relative protein abundance. MDA-MB-231 cells were used as the positive control. β-Actin was used as the loading control. (**D**) SkBr3 cells were cultured under serum-free conditions for 48 h under substrate-independent conditions on uncoated wells. Representative gelatin zymogram showing MMP9 and MMP2 activities. (**E**) SkBr3 cells were treated with various concentrations of AE cells for 24 h. Migration ability of the cells were assayed (*P < 0.01). (**F**) SkBr3 cells were treated with various concentrations of AE cells for 16 h. The invasion assay was assayed (*P < 0.05). (**G**) SkBr3 treated with various concentrations of AE for 7 days. The mammosphere-forming capacity was assayed (*P < 0.05). (**H**) Magnification, ×200; Scale bar, 20 μm. Results are expressed as the number of mammospheres per 1000 seeded cells at 5 days (mean ± SD, *n* = 3).

Twist has been demonstrated to be a major regulator of EMT [27]. We evaluated whether AE inhibited EMT and cancer metastasis in HER-2-overexpressing cell lines. Our results showed that increasing concentrations of AE significantly restored the epithelial cell adhesion protein E-cadherin and suppressed vimentin expression, a mesenchymal cell marker. MDA-MB-231, which is a highly metastatic TNBC cell line, was used as the mesenchymal cell control (Figure 5A). Immunofluorescence staining of HER-2-overexpressing cell lines after AE treatment revealed significantly increased E-cadherin expression (Figure 5B). Interestingly, treatment of HER-2-overexpressing cell lines with AE significantly reduced the cancer metastasis transcription factors Snail, Slug, Twist, and HIF-1α expression in a dose- (Figure 5C) and time-dependent (Supplementary Figure 1) manner. Malignant cells produce proteolytic enzymes, including serine proteinase, cathepsins, metalloproteinases (MMPs), and heparanase, among which MMP-9 and MMP-2 play key roles in destroying the basement membrane for mediating cancer invasion and metastasis [28]. The gelatin zymography assay confirmed that increasing concentrations of AE significantly reduced the activity of MMP-9 and MMP-2 in HER-2-overexpressing cell lines (Figure 5D). The Transwells assay showed that treatment of HER-2-overexpressing cells with increasing concentrations of AE significantly inhibited the cancer cell migration (Figure 5E) and invasion (Figure 5F) rates. We determined whether AE treatment of HER-2-overexpressing cancer cells inhibited cancer stem cells. The results showed that treatment of HER-2-overexpressing cells with increasing concentrations of AE significantly inhibited the number of mammosphere formation (Figure 5G) and the size of mammosphere formed (Figure 5H). Therefore, we concluded that AE inhibited EMT involved in cancer metastasis and cancer stem cells.

### AE inhibited ILK signaling pathways in SkBr3 cells

**Figure 6:**
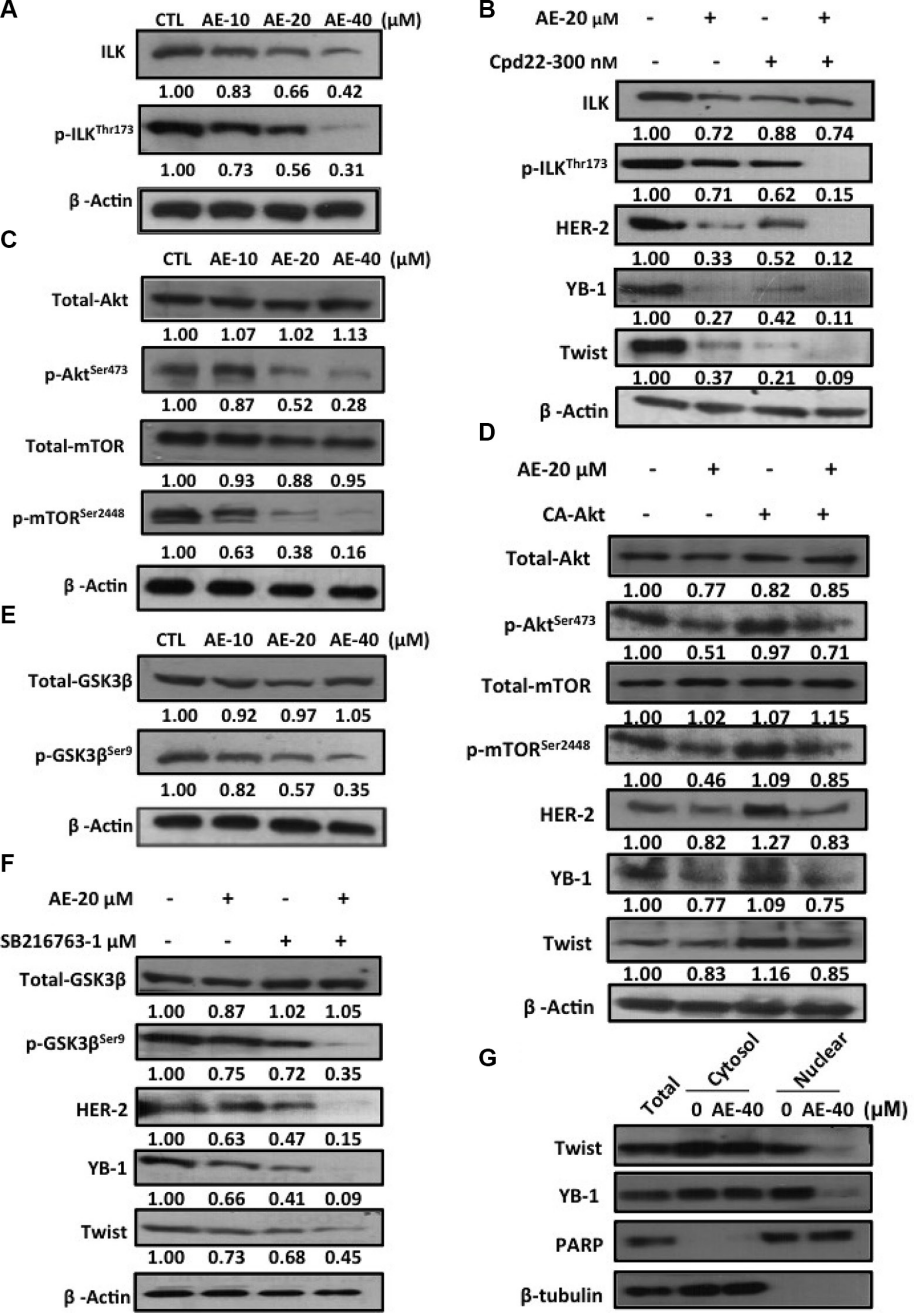
Aloe-emodin inhibited ILK signaling pathways in HER-2-overexpressing breast cancer cells (**A**) SkBr3 cells were treated with various concentrations of AE for 48 h. Cell lysates were immunoblotted with anti-ILK and anti-phospho-ILKThr173 antibody. (**B**) SkBr3 cells were treated with 20 μM AE or 300 nM ILK inhibitor cpd-22 for 48 h. Cell lysates were immunoblotted with anti-ILK, anti-phospho-ILKThr173, anti-HER-2, anti-YB-1, and anti-Twist antibodies. (**C**) SkBr3 cells were treated with various concentrations of AE for 48 h. Cell lysates were immunoblotted with anti-phospho-AktSer473 and anti-phospho-mTORSer2448 antibodies. (**D**) SkBr3 cells were transfected with constitutively active Akt and treated with 20 μM AE for 48 h. Cell lysates were immunoblotted with anti-phospho-AktSer473, anti-phospho-mTORSer2448, anti-HER-2, anti-YB-1, and anti-Twist antibodies. β-Actin was used as the loading control. (**E**) SkBr3 cells were then harvested and lysed for the detection of phospho-GSK3βSer9 and β-Actin. (**F**) SkBr3 cells were treated with 20 μM AE or 1 μM phospho-GSK3βSer9 inhibitor SB216763 for 48 h. Cell lysates were immunoblotted with anti-phospho-GSK3βSer9, anti-HER-2, anti-YB-1, and anti-Twist antibodies. (**G**) SkBr3 cells were treated with 40 μM AE for 24 h. Following cell fractionation, Twist and YB-1 content in the cytoplasmic or nuclear fraction was determined through Western blotting. PARP was used as the nuclear marker. β-Tubulin was used as the cytoplasmic marker.

A study has indicated that altering the intracellular integrin-linked kinase (ILK)/protein kinase B (Akt)/mTOR signaling pathway regulated YB-1 expression and/or cellular localization [29]. We next observed whether AE treatment inhibited the expression of ILK. The results showed that increasing concentrations of AE reduced ILK and phosphorylation of ILK at threonine 173 protein expression (Figure 6A). Next, the ILK inhibitor cdp-22 was used to treat HER-2-overexpressing breast cancer cell lines. Compared with treatment with AE or cdp-22 alone, the combined therapy more strongly inhibited phosphorylation of ILK and downstream HER-2, YB-1, and Twist protein expressions (Figure 6B). Extracellular growth factor and cell surface tyrosine receptor kinase activation resulted in PI3K activation, followed by phosphorylation of the downstream mTOR [30]. We investigated whether AE treatment inhibited the phosphorylation of Akt at serine 473 and mTOR at serine 2448. The results showed that increasing concentrations of AE reduced phosphorylation of Akt at serine 473 and mTOR at serine 2448 (Figure 6C). We confirmed these results through the use of constitutively active Akt (CA-Akt), which continued to phosphorylate Akt. The results confirmed that treatment with CA-Akt alone significantly increased Akt phosphorylation and downstream protein expression. Combining CA-Akt and AE treatment substantially restored the inhibition of AE in downstream proteins p-mTOR, HER-2, YB-1, and Twist (Figure 6D). Increased GSK3β phosphorylation has been shown to cause rapid cancer growth. Increasing concentrations of AE also inhibited GSK3β phosphorylation (Figure 6E). GSK3β inhibitor SB216763 was used to observe the effect of AE on downstream protein expression through the GSK3β pathway. The results showed that combined treatment more significantly inhibited GSK3β phosphorylation and downstream HER-2, YB-1, and Twist expressions (Figure 6F). Using nuclear/cytoplasmic separation techniques, we observed whether AE inhibited transport of transcription factors into the nucleus. The results showed that AE treatment for 24 h significantly inhibited the transport of Twist and YB-1 into the nucleus. PARP and β-tubulin were used as nucleus and cytoplasmic markers, respectively (Figure 6G). Thus, AE inhibited the expression of YB-1 through the ILK signaling pathway in HER-2-overexpressing cell lines.

### Antitumor activity of AE in SkBr3 cell xenografts model

**Figure 7:**
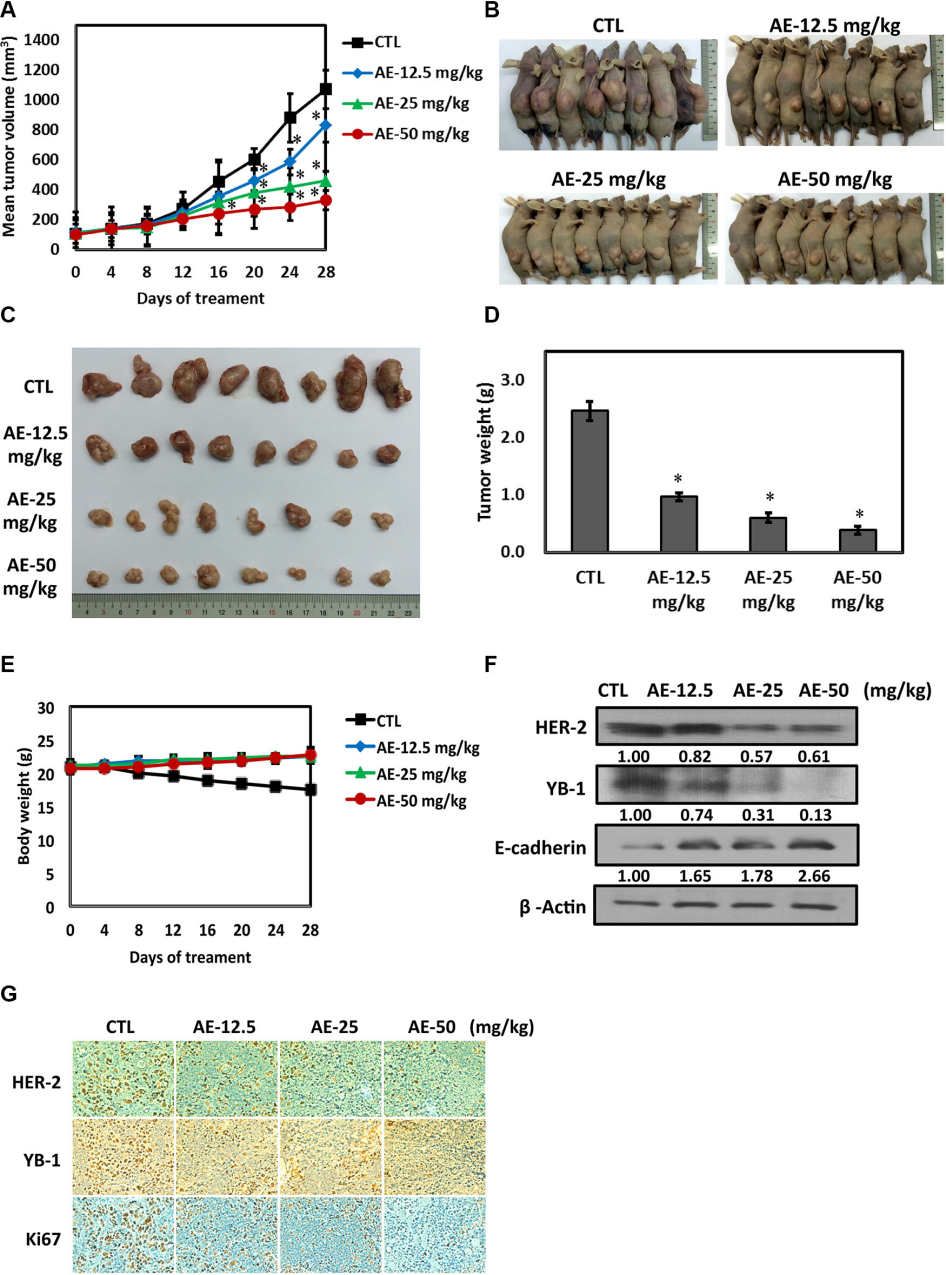
Effects of aloe-emodin on anti-tumor activity (**A**) SkBr3 cells were used to establish xenografts in male BALB/c nude mice. Animals (six mice/group) were given control and AE (12.5, 25, and 50 mg/kg) by i.p. injection 5 times for 14–18 days. Tumor size was monitored through serial caliper measurements twice a week. Each point represents mean tumor size ± SE. (**B**) One representative mouse and its tumors are shown. (**C**) Representative tumors in each group are demonstrated. (**D**) Tumor weight was calculated as indicated in Materials and methods section. (**E**) Weekly body weight measurements indicated that therapy was not toxic. Each point represents mean ± SE. (**F**) Tumor tissues were immunoblotted with anti-HER-2, anti-YB-1, and anti-E-cadherin antibodies. (**G**) Tumor tissue was collected at the conclusion of therapy, fixed in 10% normal buffered formalin, and embedded in paraffin. Four-micron (4 μM) sections of tumor tissue were assessed using immunohistochemistry for androgen receptor expression. Immunohistochemical analyses in xenograft tumors on day 28 after AE treatment were performed using antibodies against HER-2, YB-1, and Ki67. Magnification, ×40; scale bar, 500 μM.

Nude mice were injected with 3 × 10^6^ HER-2-overexpressing breast cancer cells for 14 days to induce the formation of solid tumors and were given AE once a day for 5 consecutive days, and tumor size changes were observed twice weekly. AE significantly inhibited tumor size (Figure 7A). The tumors of representative mice showed the same result (Figure 7B). The tumors were removed to observe its appearance (Figure 7C) and weight (Figure 7D); increasing AE concentrations significantly inhibited tumor size and weight. Interestingly, AE did not cause weight loss in mice. (Figure 7E). We examined HER-2, YB-1, and E-cadherin expression in the tumor specimen through Western blotting. The results confirmed that AE treatment significantly reduced HER-2 and YB-1 oncogenic protein expression and restored epithelial cell adhesion protein E-cadherin expression (Figure 7F). Similar results were clearly observed in IHC staining; AE treatment significantly reduced HER-2, YB-1, and cell proliferation marker Ki67 expression (Figure 7G). Therefore, AE treatment significantly inhibited *in vivo* tumors.

## DISCUSSION

HER-2 is found on the surface of all normal cells and receives signals for the regulation of cell growth [31]. However, in cancer cells, owing to gene amplification, many HER-2 receptor proteins are present on the surface of cells, leading to excessive growth of cells [32]. Cancer cells show rapid growth, division, and even more rapid metastasis. Compared with patients with early HER-2-overexpressing breast cancer, the probability of recurrence after resection is higher in patients with HER-2 overexpression, which accounts for approximately 25–30% of all breast cancer patients [33]. Due to the recurrence rate of HER-2-overexpressing breast cancer, early HER-2 breast cancer treatment after surgery depends on the patient's condition; they can undergo hormone therapy, chemotherapy, or radiotherapy as adjuvant treatment [34]. The target agent therapy clearly influences relapse prevention of HER-2-overexpressing breast cancer; therefore, the use of a target therapy as an adjuvant therapy is very crucial [35].

In recent years, much attention has been paid to the anti-tumor effect of emodin, with the main focus on neuroectodermal tumors, liver cancer, lung squamous cell carcinoma, merkel cell skin cancer, stomach cancer, and leukemia [36]. Emodin inhibits K562 leukemia cells and prolongs survival [37]. A possible mechanism is the inhibition of cancer cell DNA, RNA, and protein biosynthesis. AE (1,8-dihydroxy-3-(hydroxymethyl)-9,10-anthracenedione) is a bioactive anthraquinone compound extracted from rhubarb roots. It effectively inhibits the proliferation of human colon cancer cell lines and induces apoptosis [38]. However, the inhibition of growth ability and mechanism of AE in HER-2-overexpressing cell lines are unclear. Therefore, we investigated the molecular mechanisms of the anthraquinone compounds inducing apoptosis of HER-2-overexpressing human breast cancer cell lines. In particular, we compared three anthraquinones, namely emodin, AE, and rhein, for HER-2 inhibitory activity and the optimal AE concentration (Figure 1A–1D). We confirmed that AE treatment mainly reduced cancer cell viability by inducing apoptosis (Figure 2H–2J).

Recent studies have shown that various characteristics of cancer stem cells, such as their high mobility, immune specificity, and metabolic specificity, differ considerably from our understanding of cancer cells; therefore, the development of cancer stem cell treatment is the future of cancer treatment [39–41]. The multifunctional protein YB-1 has been found in many cancers, with the common characteristics of promotion of stem cell marker expression, improvement of mobility, and enhancement of cancer drug resistance gene expression [42–45]. Therefore, the inhibition of YB-1 expression has attracted much attention. In this study, we confirmed that AE treatment significantly reduced YB-1 expression in HER-2-overexpressing cell lines (Figure 3).

EMT is a key event in cancer metastasis. Cancer metastasis is directly related to Twist gene transfection during cancer cell hypoxia [46]. Hypoxia of head and neck cancer most likely occurs if the transfection of the hypoxic gene HIF-1α also results in the transfer of Twist and Snail genes, resulting in the worst prognosis in patients [47]. Hypoxia gene HIF-1α overexpression can cause lung metastases in mice [48, 49]. Simultaneous expression of hypoxia gene HIF-1α in head and neck cancer tumor tissues promotes the expression of Twist and Snail genes, leading to the worst prognosis [50, 51]. This study confirmed that AE treatment significantly inhibited the oncogenic Twist expression (Figure 4). AE treatment significantly restored epithelial cell marker protein E-cadherin, inhibited the stromal cell marker protein vimentin, and suppressed EMT-associated transcription factors, including Twist, Snail, Slug, and HIF-1α, inhibiting EMT induction in cancer cells. Furthermore, we confirmed that AE significantly inhibited HER-2-overexpressing breast cancer cell migration and invasion. Interestingly, the stem cell experiments confirmed that AE effectively inhibited the growth of stem cells (Figure 5).

The PI3K/Akt/mTOR signal transduction pathway can regulate gene expression and is crucial in breast tumor cell growth proliferation, metastasis, and apoptosis [52, 53]. ILK kinase activity and expression affect the activity of MMP-2 and MMP-9 [54, 55]. Studies have indicated that the inhibition of Akt activity leads to decreased activity of MMP-2 and MMP-9 (Figure 5D). This study confirmed that increasing concentrations of AE significantly inhibited the PI3K/Akt/mTOR signal transduction pathway. In particular, AE inhibited the oncogenic transcription factors Twist and YB-1 from entering the nucleus, thereby inhibiting downstream oncogenic protein expression (Figure 6).

In the xenograft model, SkBr3 cells were subcutaneously injected to BALB/c nude mice and successfully induced subcutaneously tumors in BALB/c nude mice. Notably, compared with the control group, AE treatment for 28 days caused 80% inhibition of tumor growth; weight loss did not occur, thus confirming its low toxicity. Moreover, AE inhibited HER-2 and YB-1 expression in tumor tissue and restored E-cadherin expression (Figure 7).

**Figure 8:**
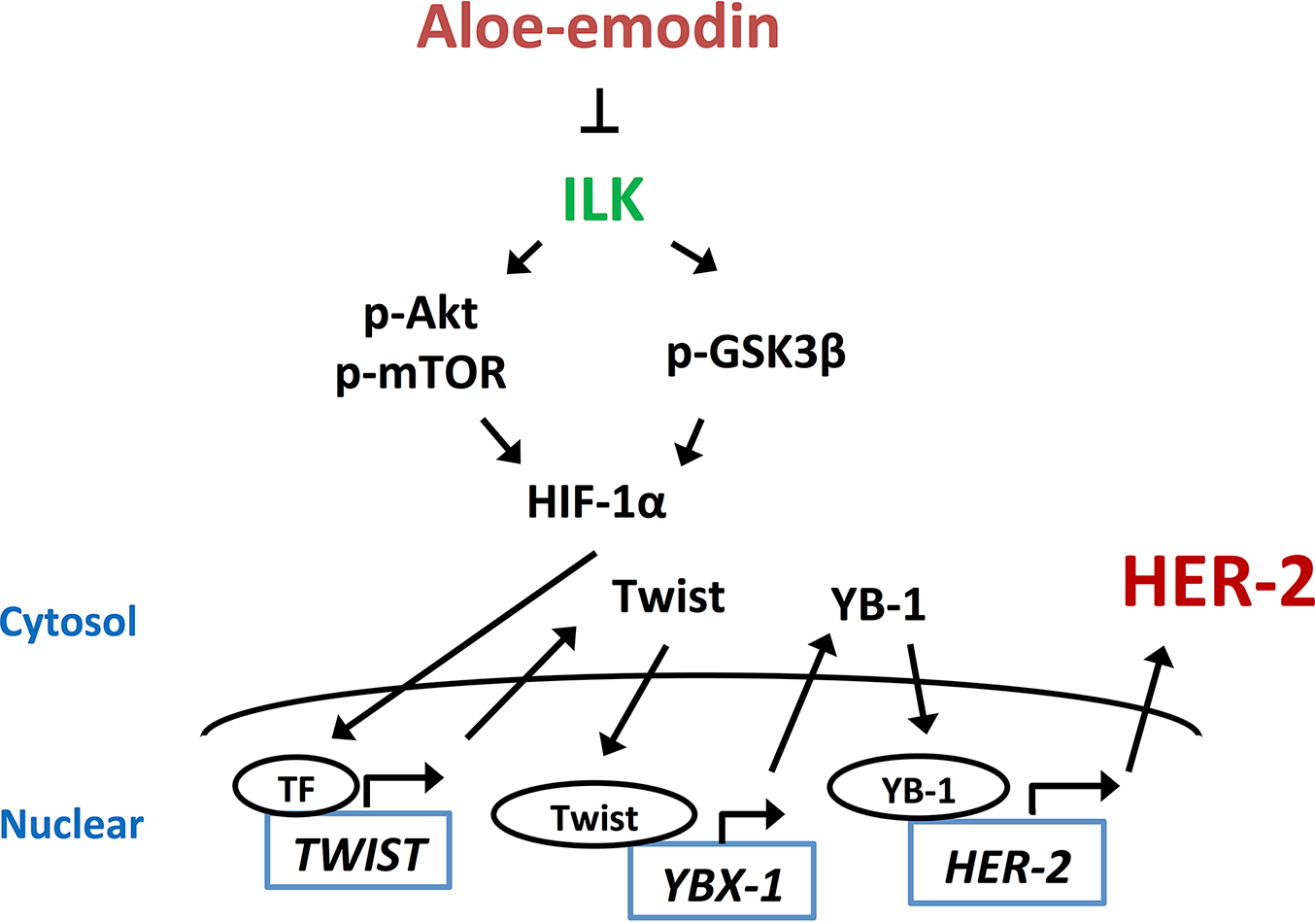
The signaling pathways of Aloe-emodin inhibits HER-2 expression

In summary, the results indicated that AE inhibited the expression of HER-2 through the downregulation of YB-1. AE suppressed YB-1 expression through the downregulation of ILK/Akt/mTOR-regulated Twist expression in HER-2-overexpressing breast cancer cells (Figure 8). Importantly, AE treatment could be used for clinically treating patients with HER-2 overexpression in the future.

## MATERIALS AND METHODS

### Materials

Cell culture materials were obtained from Invitrogen (Burlington, Ontario, Canada). The reagents 3-(4,5-dimethylthiazol-2-yl)-2,5-diphenyl tetrazolium bromide (MTT), propidium iodide (PI) and the PureLink™ HiPure Plasmid DNA Purification Kit were purchased from Sigma (St. Louis, MO, USA). Primary antibodies against PARP and Twist were purchased from GeneTex (Beverly, MA, USA). Anti-HER-2, anti-phospho-Akt (Ser473), anti-E-cadherin, anti-vimentin, anti-Snail, and anti-Slug antibodies were purchased from Cell Signaling (Beverly, MA, USA). Primary antibodies against ILK, phosphor-ILK (Thr173), phospho-mTOR (Ser2448), and phospho-GSK3β (Ser9) were purchased from Santa Cruz Biotechnology (Santa Cruz, CA, USA). Antibodies against YB-1 and β-Actin were purchased from Millipore (Temecula, CA, USA). The antibody against HIF-1α was purchased from BD Biosciences Clontech (San Jose, CA, USA). For Western blotting, the secondary antibodies of horseradish peroxidase (HRP)-conjugated goat anti-mouse IgG and goat anti-rabbit IgG were obtained from Millipore (Temecula, CA, USA), and enhanced chemiluminescence (ECL) reagents were purchased from Sigma-Aldrich. Emodin, AE, and rhein were purchased from Sigma-Aldrich. The RNAi Consortium of *YBX-1* was selected by the National RNAi Core Facility.

### Cell culture

Human breast cancer cell lines SkBr3, BT-474, MDA-MB-453, MCF-7, BT-20, MDA-MB-231 and human breast cell MCF-10A were obtained from the American Type Culture Collection (Manassas, VA, USA). Human breast cancer cell lines were cultured in Dulbecco's modified Eagle's medium (DMEM)/F12 and Roswell Park Memorial Institute (RPMI 1640) (Invitrogen, Carlsbad, CA, USA) supplemented with 10% fetal bovine serum (FBS) and 1% penicillin-streptomycin. Human breast cell MCF-10A was cultured in Dulbecco's modified Eagle's medium (DMEM)/F12 supplemented with 5% horse serum, Insulin 10 μg/mL, EGF 20 ng/mL and 1% penicillin-streptomycin. Cells were incubated at 37°C in an incubator containing 5% CO_2._

### MTT assay

Cells were plated at 2 × 10^4^ cells per well in triplicate in 24-well plates and incubated in a medium containing 10% FBS. After 24 h of incubation, the complete medium was replaced with the test medium containing vehicle control or various doses of emodin, AE, and rhein for 24, 48, and 72 h at 37°C. MTT solution (stock concentration: 5 mg/mL in PBS) was diluted to 500 μg/mL. MTT working solution (100 μL) was added to each well and incubated at 37°C for 2 h. The absorbance was measured at 590 nm by using a 96-well plated reader.

### Cell cycle and annexin V-PI analysis

Cell cycle and Annexin V-PI analysis were conducted as previously described [56].

### Western blotting

Cells in 10-cm culture dishes (1 × 10^6^ per dish) were treated with the indicated drugs. After treatment, total proteins were extracted by adding 50 μL cold lysis buffer (50 mM Tris-HCl, pH 7.4; 1mM NaF; 150 mM NaCl; 1 mM EGTA; 1 mM phenylmethylsulfonyl fluoride; 1% NP-40; and 10 mg/mL leupeptin) to the cell pellets overnight at −20°C, followed by centrifugation at 12 000 × *g* for 30 minutes. Western blotting was conducted as recently described [57].

### Cell transfection

Cells were transfected with 50 nmol/L YBX-1-siRNA and cDNA-HER-2 using Oligofectamine (Invitrogen, Carlsbad, CA, USA) in a serum-free medium. Six hours after transfection, the medium was replaced with a medium supplemented with 10% FBS. After a 24-h transfection, medium was replaced with a medium supplemented with 10% FBS with or without AE (10 or 20 μM), and the cells were incubated for 48 h. After harvesting, the cells were lysed and prepared for Western blotting.

### Immunofluorescence

Expression of HER-2, YB-1, Twist, and E-cadherin in the cells was analyzed through Leica confocal microscopy conducted using a monoclonal primary antibody [58].

### Colony formation assay

SkBr3 cells were seeded into six-well plates with 2 mL culture medium containing 10% FBS. The colonies were counted after culturing in DMEM-F12 containing 10% FBS at 37°C in a humidified, 5% CO_2_ atmosphere. SkBr3 cells were washed twice with PBS, stained with Giemsa, and colonies with > 50 cells were counted.

### Soft agar assay

SkBr3 cells were treated with 40 μM emodin, AE, and rhein or various doses of AE. Cells were suspended in a medium containing 0.4% agar and overlaid on 1% agar in 24-well plates (500 cells per well), respectively. After 2–3 weeks, colonies were counted and photographed.

### Migration assay

Cell monolayers were wounded using a sterile pipette tip and rinsed with PBS to remove cellular debris. Cells were treated with various doses of AE for 24 h. The phase contrast images of the wounds were obtained at 37°C for incubation for 0 and 24 h, and three separate experiments were performed.

### Invasion assays

SkBr3 cell invasion assays were performed in 6.5-mm Transwell chambers (8.0-μm pore size) (Coring Incorporation, NY, USA), and the polycarbonate filters were coated with diluted matrigel (BD Biosciences). SkBr3 cells without and with AE were added to the coated filters (6 × 10^4^ cells per filter) in 200 μL serum-free DMEM-F12 in triplicate wells. DMEM-F12 medium containing 10% FBS was added to the lower chambers. After 24-h incubation at 37°C in a 5% CO_2_ and 95% humidity incubator, the cells that did not migrate and remained on the upper surface of the filter; they were wiped off using a cotton swab. Migrated cells were fixed in 95% alcohol for 10 minutes and stained with Giemsa for 10 minutes.

### Gelatin zymography assay

Gelatin zymography was performed on protein extracts from the SkBr3 cells. In brief, the cells were rinsed three times with DPBS and cultured in 3 mL serum-free DMEM-F12 for 24 h. The cells without and with AE were separated on 12.5% SDS-PAGE containing 1 mg/mL gelatin. Following electrophoresis at 48 °C, the gel was soaked in 2.5% Triton X-100 to remove SDS, rinsed three times in H2O, transferred to a bath containing 0.1 M glycine-NaOH (pH 8.3), and incubated at 37°C for 16 h. Subsequently, the gel was fixed, stained with 0.5% Coomassie blue in 30% isopropanol and 10% acetic acid for 1 h, and destained in 12.5% isopropanol and 10% acetic acid.

### Quantitative PCR assay

RNA was isolated using the RNeasy^®^ Plus Mini Kit (Qiagen, Valencia, CA). cDNA was amplified using gene-specific primers and the Power SYBR Green PCR Master Mix (Applied Biosystems). Reverse transcription of mRNA was performed using the RevertAid First Strand cDNA Synthesis Kit (Thermo Scientific) according to manufacturer instructions. mRNA expression was normalized to human *YBX-1* and *TWIST1* expression. The results are presented as the relative fold expression compared with the respective control treatmen^t.^

### Nuclear extraction

The cells were harvested, washed with PBS, and centrifuged at 700 × *g* for 5 minutes; the supernatant was discarded. Cell pellets were first prepared in cytosolic extraction buffer with protease inhibitor and dithiothreitol (DTT) and centrifuged at 16 000 × *g* for 10 minutes, and the supernatant was extracted as cytosolic fractions. Subsequently, cell pellets were prepared in NER buffer with protease inhibitor and DTT. After vortexing 4 times for 15 seconds, the cells were centrifuged at 16 000 × *g* for 10 minutes, and the supernatant was extracted as nuclear fractions. All experiments were performed using the Nuclear/Cytosol Fractionation Kit (BioVision, Milpitas, CA, USA) according to manufacturer instructions. Proteins in the cytosolic and nuclear fractions were quantified through Bradford assays (Bio-Rad), and protein expression was measured through Western blotting.

### *In vivo* studies

Female BALB/c nude mice (18–20 g; 6–8 weeks of age) were purchased from the National Animal Center (Taipei, Taiwan) and maintained in pressurized ventilated cage in accordance with institutional regulations. SkBr3 cells (3 × 10^6^) were inoculated subcutaneous into the right flank of the mice. Seven days after inoculation, when tumor volumes were larger than 100 mm^3^, the mice were divided into four groups (six mice per group) and treated daily with vehicle alone and various doses of AE (12.5, 25, and 50 mg/kg). The mice were weighed, and their tumors were measured using calipers twice per week by using a digital caliper. The tumor volume was calculated using the following formula: (width × length^2^)/2. On the final day of treatment, the mice were sacrificed; the tumors were excised, weighed, and sectioned; the tumor sections were embedded in an optimal cutting temperature (OCT) compound and frozen at −70°C.

### Immunohistochemical staining

Sections frozen in OCT were fixed in acetone and chloroform. After overnight incubation with primary antibodies, including mouse monoclonal anti-HER-2, anti-YB-1, and anti-E-cadherin, the slides were washed again with Tris-buffered saline and Tween20 and incubated with biotinylated secondary antibodies and subsequently with the avidin–biotin–horseradish peroxidase complex (Vector Laboratories, San Mateo, CA, USA). Antibody detection was performed using 3,3′-diaminobenzidine, and the tissue sections were counterstained with Mayer's hematoxylin, washed, mounted with Universal Mount, and dried on a 56°C hot plate. The prepared slides were examined through light microscopy.

### Statistical analyses

All values are expressed as mean ± SD. Each value is the mean of at least three separate experiments in each group. The two-tailed Student *t* test was used to compare the continuous variables between the two groups (*P < 0.05; **P < 0.01; ***P < 0.001).

## SUPPLEMENTARY MATERIALS FIGURE


