# Correction: Aloe-emodin inhibits HER-2 expression through the downregulation of Y-box binding protein-1 in HER-2-overexpressing human breast cancer cells

**DOI:** 10.18632/oncotarget.27230

**Published:** 2019-10-01

**Authors:** Jui-Wen Ma, Chao-Ming Hung, Ying-Chao Lin, Chi-Tang Ho, Jung-Yie Kao, Tzong-Der Way

**Affiliations:** ^1^ Institute of Biochemistry, College of Life Science, National Chung Hsing University, Taichung, Taiwan; ^2^ Department of General Surgery, E-Da Hospital, I-Shou University, Kaohsiung, Taiwan; ^3^ School of Medicine, I-Shou University, Kaohsiung, Taiwan; ^4^ Division of Neurosurgery, Buddhist Tzu Chi General Hospital, Taichung Branch, Taiwan; ^5^ School of Medicine, Tzu Chi University, Hualien, Taiwan; ^6^ Department of Medical Imaging and Radiological Science, Central Taiwan University of Science and Technology, Taichung, Taiwan; ^7^ Department of Food Science, Rutgers University, New Brunswick, New Jersey, USA; ^8^ Department of Biological Science and Technology, College of Biopharmaceutical and Food Sciences, China Medical University, Taichung, Taiwan; ^9^ Department of Health and Nutrition Biotechnology, College of Health Science, Asia University, Taichung, Taiwan

**This article has been corrected:** Due to errors in figure preparation, the Western ink point p-Akt protein was accidentally used in the wrong picture in Figure 6D. In addition, in Figure 7G, the treatment AE 25 mg/kg group HER2 protein is also incorrect due to erroneous group photos up to AE 50 mg/kg. The authors declare that these corrections do not change the results or conclusions of this paper.

Original article: Oncotarget. 2016; 7:58915–58930. 58915-58930. https://doi.org/10.18632/oncotarget.10410

**Figure 6:**
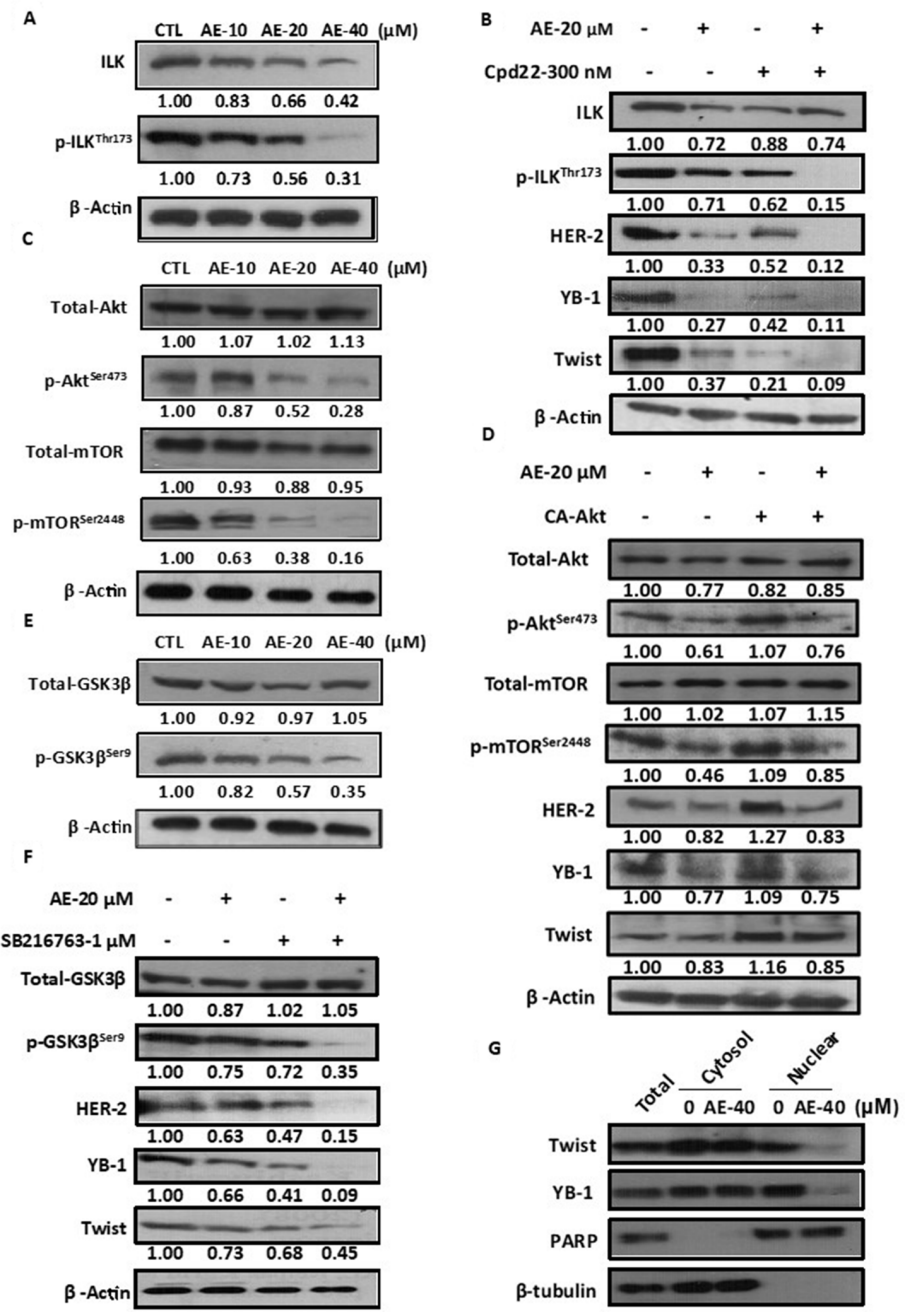
Aloe-emodin inhibited ILK signaling pathways in HER-2-overexpressing breast cancer cells. (**A**) SkBr3 cells were treated with various concentrations of AE for 48 h. Cell lysates were immunoblotted with anti-ILK and anti-phospho-ILKThr173 antibody. (**B**) SkBr3 cells were treated with 20 μM AE or 300 nM ILK inhibitor cpd-22 for 48 h. Cell lysates were immunoblotted with anti-ILK, anti-phospho-ILKThr173, anti-HER-2, anti-YB-1, and anti-Twist antibodies. (**C**) SkBr3 cells were treated with various concentrations of AE for 48 h. Cell lysates were immunoblotted with anti-phospho-AktSer473 and anti-phospho-mTORSer2448 antibodies. (**D**) SkBr3 cells were transfected with constitutively active Akt and treated with 20 μM AE for 48 h. Cell lysates were immunoblotted with anti-phospho-AktSer473, anti-phospho-mTORSer2448, anti-HER-2, anti-YB-1, and anti-Twist antibodies. β-Actin was used as the loading control. (**E**) SkBr3 cells were then harvested and lysed for the detection of phospho-GSK3βSer9 and β-Actin. (**F**) SkBr3 cells were treated with 20 μM AE or 1 μM phospho-GSK3βSer9 inhibitor SB216763 for 48 h. Cell lysates were immunoblotted with anti-phospho-GSK3βSer9, anti-HER-2, anti-YB-1, and anti-Twist antibodies. (**G**) SkBr3 cells were treated with 40 μM AE for 24 h. Following cell fractionation, Twist and YB-1 content in the cytoplasmic or nuclear fraction was determined through Western blotting. PARP was used as the nuclear marker. β-Tubulin was used as the cytoplasmic marker.

**Figure 7:**
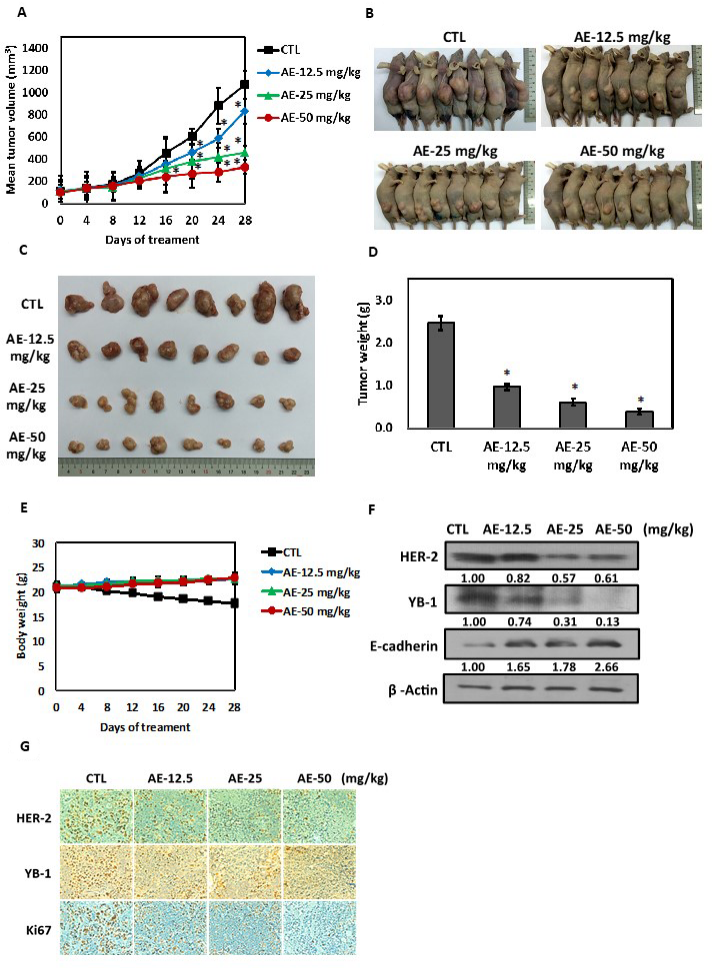
Effects of aloe-emodin on anti-tumor activity. (**A**) SkBr3 cells were used to establish xenografts in male BALB/c nude mice. Animals (six mice/group) were given control and AE (12.5, 25, and 50 mg/kg) by i.p. injection 5 times for 14–18 days. Tumor size was monitored through serial caliper measurements twice a week. Each point represents mean tumor size ± SE. (**B**) One representative mouse and its tumors are shown. (**C**) Representative tumors in each group are demonstrated. (**D**) Tumor weight was calculated as indicated in Materials and methods section. (**E**) Weekly body weight measurements indicated that therapy was not toxic. Each point represents mean ± SE. (**F**) Tumor tissues were immunoblotted with anti-HER-2, anti-YB-1, and anti-E-cadherin antibodies. (**G**) Tumor tissue was collected at the conclusion of therapy, fixed in 10% normal buffered formalin, and embedded in paraffin. Four-micron (4 μM) sections of tumor tissue were assessed using immunohistochemistry for androgen receptor expression. Immunohistochemical analyses in xenograft tumors on day 28 after AE treatment were performed using antibodies against HER-2, YB-1, and Ki67. Magnification, ×40; scale bar, 500 μM.

