# Supplementary material for: Aloe-emodin inhibits HER-2 expression through the downregulation of Y-box binding protein-1 in HER-2-overexpressing human breast cancer cells
Source: Oncotarget. 2016 Jul 6;7(37):58915–30. doi: 10.18632/oncotarget.10410 (PMC5312285; doi:10.18632/oncotarget.10410)
Supplement: Supplementary file 1 [file oncotarget-07-58915-s001.pdf]

Aloe-emodin inhibits HER-2 expression through the downregulation of Y-box binding protein-1 in HER-2-overexpressing human breast cancer cells

Supplementary Materials

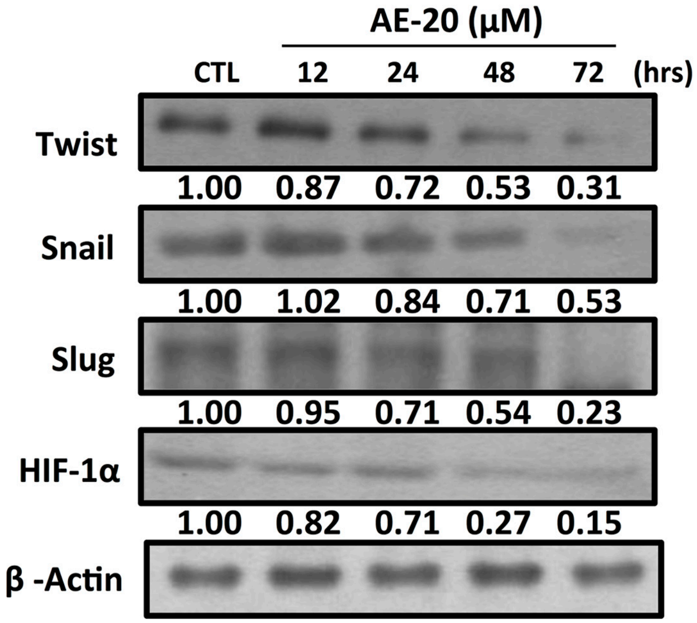

Supplementary Figure S1: SkBr3 cells treated with various times of Aloe-emodin (AE) 20 μM, the expression of transcription factor Twist, Snail, Slug, and HIF-1α were assayed by Western blotting. Values represent relative protein abundance. β-Actin was used as the loading control.
